# A mathematical model of seropositivity to malaria antigen, allowing seropositivity to be prolonged by exposure

**DOI:** 10.1186/1475-2875-13-12

**Published:** 2014-01-08

**Authors:** Samuel Bosomprah

**Affiliations:** 1 Department of Biostatistics, School of Public Health, University of Ghana, Legon, Accra, Ghana

## Abstract

**Background:**

Malaria transmission intensity is traditionally estimated from entomological studies as the entomological inoculation rate (EIR), but this is labour intensive and also raises sampling issues due to the large variation from house to house. Incidence of malaria in the control group of a trial or in a cohort study can be used but is difficult to interpret and to compare between different places and between age groups because of differences in levels of acquired immunity. The reversible catalytic model has been developed to estimate malaria transmission intensity using age-stratified serological data. However, the limitation of this model is that it does not allow for persons to have their seropositivity boosted by exposure while they are already seropositive. The aim of this paper is to develop superinfection mathematical models that allow for antibody response to be boosted by exposure.

**Method:**

The superinfection models were fitted to age-stratified serological data using maximum likelihood method.

**Results:**

The results showed that estimates of seroconversion rate were higher using the superinfection model than catalytic model. This difference was milder when the level of transmission was lower. This suggests that the catalytic model is underestimating the transmission intensity by up to 31%. The duration of seropositivity is shorter with superinfection model, but still seems too long.

**Conclusion:**

The model is important because it can produce more realistic estimates of the duration of seropositivity. This is analogous to Dietz model, which allowed for superinfection and produced more realistic estimates of the duration of infection as compared to the original Ross-MacDonald malaria model, which also ignores superinfection.

## Background

Measurement of malaria transmission intensity is important for several reasons. It can be used to assess the impact of public health measures on transmission and to understand epidemiological patterns, such as the age distribution of malaria illness. It can also be used for planning intervention studies and interpreting their results. Malaria transmission intensity is traditionally estimated from entomological studies as the entomological inoculation rate (EIR) but this is labour intensive and also raises sampling issues due to the large variation from house to house. Incidence of malaria in the control group of a trial or in a cohort study can be used but is difficult to interpret and to compare between different places and between age groups because of differences in levels of acquired immunity. For common viral infections such as measles, persons can be classed simply as either susceptible or immune, and the force of infection estimated from incidence data, allowing for the fact that individuals become immune after an infection, an approach first developed by [1] who introduce the catalytic model. For common viral diseases such as measles the presence of antibodies indicates the person has been infected and is immune to subsequent infection, the force of infection can then be estimated directly from antibody prevalence by age in cross-sectional serological surveys. This approach has been used to estimate the force of infection for measles and other infections [2-4], using an extension of Muench’s method, which allows that the force of infection may vary with age, but assuming that immunity is lifelong.

Drakeley *et al.*[5] have developed the use of serological data for estimating malaria transmission intensity, using the concentration of antibodies to MSP1_19_ measured in surveys of all age groups. They employed a reversible catalytic model, which assumes a constant rate of seroconversion (SCR) and assumes a constant rate of reversion to the seronegative state, independent of the level of transmission. They fitted this model to age-stratified serological data from 12 sites in Tanzania with varying entomological inoculation rate. The model was constrained to fit a single value for the annual rate of reversion to the seronegative state, which was estimated as 0.01393 per year, suggesting that antibodies persist for an average time of 72 years. The authors discussed possible reasons for this long duration. One possible further contributory factor for the estimated long duration is that the model does not incorporate boosting. In high transmission areas, exposure while seropositive prolongs the seropositive state. This feature can be incorporated, allowing each exposure to infection to prolong the seropositive state using a model of superinfection. The aim of this paper is to develop a mathematical model of seropositivity to malaria antigens that allows seropositivity to be prolonged by exposure. The model was validated against the field data used in Drakeley *et al.*[5]. It was also validated against field data from Bioko, where a change in transmission has been established [6].

## Methods

### Ethical statement

Ethical approval was obtained from the institutional review boards of the National Institute of Medical Research of Tanzania, Kilimanjaro Christian Medical Centre, and the London School of Hygiene and Tropical Medicine [5].

### Data source

Two datasets have been used for assessing the malaria transmission intensity. The study design and methods of data collection are similar. The first dataset involved serum samples collected on 250 people in each of 12 villages in three transects (North Pare, South Pare, and West Usambara) with different transmission intensities in Tanzania. The study design has been described in detail elsewhere [5]. The second dataset is a subset of serum samples from the 5th BIMCP survey in 2008 in 18 sentinel sites in Bioko, which was aimed at evaluating malaria intervention using serological measures. The 18 sentinel sites have been grouped into 5 based on geography for purposes of analysis. Group 3 involving three sentinel sites (Rebola, Bakake and Baney) where interventions have seen the most success was identified as the most obvious to model in terms of change in malaria transmission. The study design has been described in detail elsewhere [6].

### Statistical analysis

#### The superinfection models

**Figure 1:**
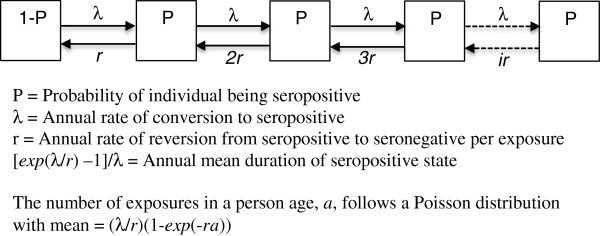
Compartmental superinfection model.

The concept of superinfection has been used to describe periods of infection prolonged by repeated exposure to infection [7-10]. Persons in endemic areas often have pre-existing partial immunity. But when these persons are removed from exposure the immunity can be lost gradually. When the person is re-exposed while seropositive the level of antibody response can be boosted. A simple way to allow for the antibody response to be boosted by exposure is to consider that each exposure gives rise to an antibody response. This can be thought of as a set of antibody-producing cells that are triggered by the exposure. Suppose the random variable *v* represents the number of such sets of cells, and x_*i*_ is the probability P(*v* = *i*). For every exposure the value of v has a one-unit increase, and when any of the sets of cells dies the value of v has a one-unit decrease. If the average duration of a set of cells is 1/*r*, the rate for the transition from *v* = *i* to *v* = *i*-1 is the product of *i* and *r* (i.e. *i*x*r*). Because there are *i* sets of cells the value of *v* reverts from *i* to *i*-1 when any of the *i* sets of cells dies off (see Figure 1).

The compartmental model (Figure 1) can be represented in differential equation as shown in equation (1) below.

(1) $$\begin{array}{l} \frac{\;dx_{i}}{\mathrm{da}}=-\lambda x_{i}+\lambda x_{i-1}-\mathrm{ir}x_{i}+\left(i+1\right)rx_{i+1},i\\ \;=0,\ldots ,\infty ;x_{i}=0\;\mathrm{for}\;i<0 \end{array}$$

where xi = Probability (number of exposures, v = *i*), so that $\sum_{i=0}^{\infty }x_{i}=1$.

These equations can be solved by a standard method, using generating functions [11].

The basic superinfection model with seroconversion rate, λ, assumed constant over time has been derived as:

(2) $$P\left(a\right)=1-\exp\left(-\frac{\lambda }{r}\left(1-\exp\left(-\mathrm{ra}\right)\right)\right)$$

where *r* is the annual rate of reversion from seropositive to seronegative state per exposure. It follows that the number of exposures in a person aged, *a* follows a Poisson distribution with mean:

(3) $$\mu =\frac{\lambda }{r}\left(1-\exp\left(-\mathrm{ra}\right)\right)$$

Suppose that λ has changed abruptly from λ_1_ to λ_2_ at a certain point in calendar time, *μ*, but is otherwise constant for different ages (i.e. λ(t) = λ_1_ (t < μ), λ(t) = λ_2_ (t ≥ μ)) , and people were observed at time *t* = *μ* +c, where c > 0. The seroprevalence at age *a* at time *t* is therefore given by:

(4) $$\begin{array}{c} P\left(t,a\right)=1-\exp\;\left[-\left\{\left(\frac{\lambda_{1}}{r}\left(\exp\left[-r\left(t-\mu \right)\right]-\exp\left[-\mathrm{ra}\right]\right)\right)\;f\left(t-a\right)\right)\right)\\ \;\left(\left(+\frac{\lambda_{2}}{r}\left(1-\exp\left[-r\left(t-\mu \right)\right]\right)\right\}\right] \end{array}$$

This is the superinfection model with an abrupt change in seroconversion rate, λ. This model can be used to investigate abrupt changes in malaria transmission in the recent time past.

#### Estimation of parameters

In the specification of the basic superinfection model there are two main parameters (λ, *r*). But in the specification of the superinfection model, which allowed an abrupt change in seroconversion rate there are three main parameters (λ_1_, λ_2_, *r*). The model was fitted to age-stratified serological data using the method of maximum likelihood. In this model, the dependent variable Y is an indicator variable, meaning that it takes on only the values 0 (= seronegative state) or 1 (= seropositive state). The probability mass function from the Bernoulli distribution - the distribution for the random indicator variable - is:

$$f\left(y_{j};\pi_{j}\right)=\left\{\begin{array}{c} \pi_{j}\;\mathrm{if}\;y_{j}=1\\ 1-\pi_{j}\;\mathrm{if}\;y_{j}=0 \end{array}\right)$$

where  0 ≤ π_j_ ≤ 1

and π_j_ was identified as the probability for a success (arbitrarily y_j_ = 1 is called a success).

The log-likelihood function for the j^th^ observation is:

$$\ln\ell_{j}=\left\{\begin{array}{c} \ln\left(P\left(\alpha_{j}\right)\right)\;\mathrm{if}\;y_{j}=1\\ \ln\left(1-P\left(\alpha_{j}\right)\;\right)\;\mathrm{if}\;y_{j}=0 \end{array}\right)$$

where y_j_ is the indicator variable y_j_ = 1 if person j is seropositive and y_j_ = 0 if they are seronegative, and *a*_j_ is their age and P(*a*_j_) is the proportion seropositive at age *a*_j_.

The antibody titre was used to classify individuals as seropositive (or responder) or seronegative (or non-responder) using the mixture model method [12]. Briefly, the distribution of normalized optical density (OD) values was fitted as the sum of two Gaussian distributions - a narrow distribution of seronegatives and a broader distribution of seropositives - using maximum likelihood methods. The mean OD of the Gaussian corresponding to the seronegative population plus three standard deviations was used as the cut-off for seropositivity [6]. A separate cut off was generated for each antigen, say MSP1 & AMA1.

The seroconversion rate (SCR) was then estimated by fitting the superinfection model to the observed seroprevalence data, stratified into yearly age groups, using the maximum likelihood methods. All members of the population become susceptible at a certain age. This is the age when maternal malaria immunity has sufficiently waned to make the children susceptible to infection. This age is an unknown constant, which can be dealt with using several approaches. Remme *et al.*[4] estimated this age as part of the model specification. Drakeley *et al.*[5] excluded children below one year and fitted the model to all villages simultaneously, allowing SCR to vary among villages but with the reversion rate constrained to a single value. Drakeley’s approach has been adopted to fit the superinfection model. The maximum likelihood estimation of the SCR has been executed using the ‘ml’ command in Stata for Windows (College Stations, Texas, USA). Briefly, the log-likelihood function of proportion seropositive at age, ***a***, and its first derivatives was evaluated using the Method-d1 evaluators [13]. Ado-files were then written to maximise the likelihood and predict the SCR using the observed age-stratified seroprevalence data.

## Results

**Figure 2:**
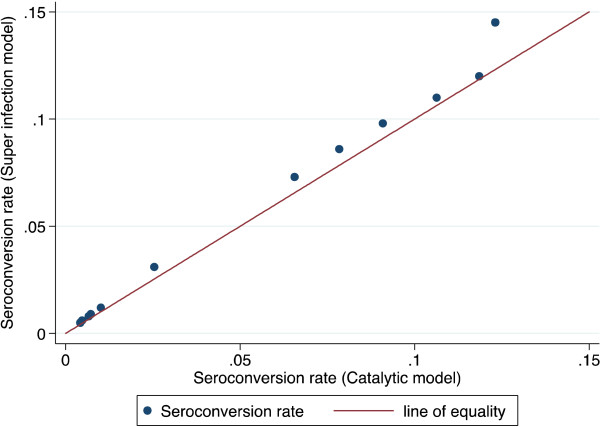
**Comparison of superinfection estimates and catalytic estimates for each village, original data used by Drakeley *et al., *** **2005, Tanzania.**

**Figure 3:**
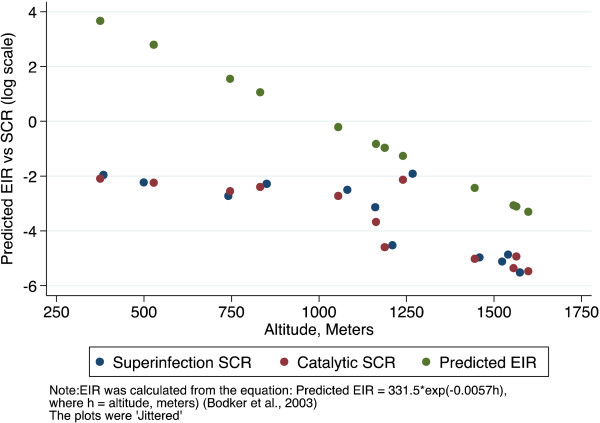
**Association between altitudes and annual rate of seroconversion from MSP1**_**19 **_**seronegative to seropositive (Superinfection & Catalytic models) or predicted EIR (Bodker *et al., *** **2003) for the 12 sites in Tanzania.**

**Figure 4:**
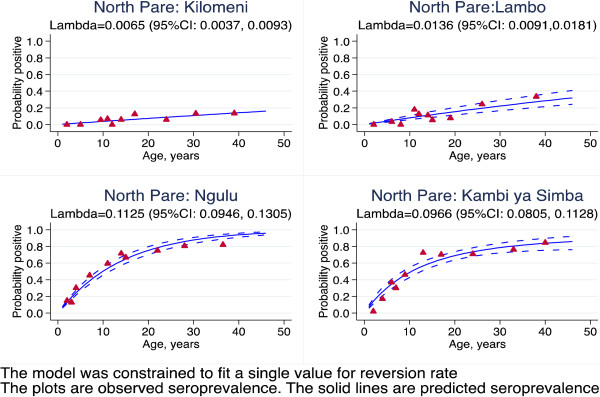
**Superinfection model of seropositivity to MSP1**_**19 **_**for villages in North Pare, Tanzania.**

**Figure 5:**
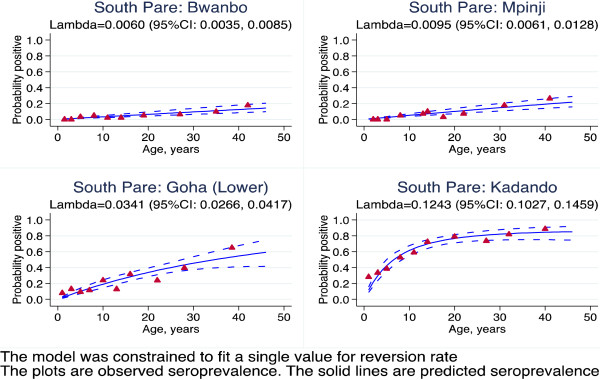
**Superinfection model of sero-positivity to MSP1**_**19 **_**for villages in South Pare, Tanzania.**

**Figure 6:**
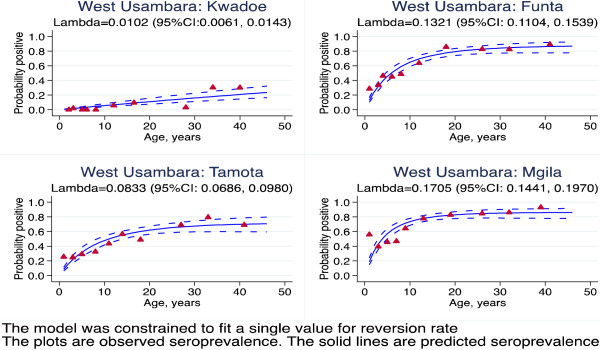
**Superinfection model of sero-positivity to MSP1**_**19 **_**for villages in West Usambara, Tanzania.**

**Figure 7:**
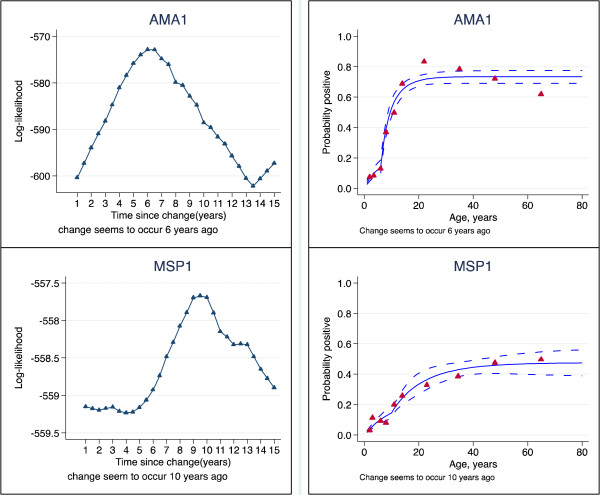
**Log-likelihood profiles and superinfection models of probability seropositive to AMA1 & MSP1**_**19 **_**, allowing abrupt change in incidence rate (Rabola,Bakake, Baney), Bioko, 2008.**

The superinfection model was compared with the original data from Tanzania. The results showed that estimates of seroconversion rate were higher using the superinfection model than catalytic model (Figure 2). This difference was milder when the level of transmission was lower. This suggests that the catalytic model might be underestimating the transmission intensity by up to 31% (Table 1). The duration is shorter with superinfection model, but still seems too long (Table 1). This may be because the model assumes a constant reversion rate. The estimates of seroconversion rate were lower than the predicted entomological inoculation rate (Figure 3). It may be that the predictive model is no longer valid. The seroprevalence profiles appeared to suggest that the model fits the seroconversion rate quite well but it may be difficult to discriminate the model on the basis of fit alone (Figures 4, 5 and 6). The superinfection model was also fitted to data from Bioko (Table 2) where a change in transmission has been established using the catalytic model. The superinfection model predicts a change in transmission, which is consistent with the catalytic model (Figure 7 and Table 3). The model, which allowed an abrupt change in seroconversion rate were better fit than the model, which did not assume that seroconversion rate has changed (Likelihood ratio test for AMA1: LR chi2 (1) = 60.11; P-value < 0.0001; and for MSP1_19_: LR chi2 (1) = 3.09; P-value = 0.08).

**Table 1:** **Comparison of estimates of seroconversion rate (SCR) and duration of seropositivity to MSP1**_**19 **_**using catalytic and superinfection models fitted to data from 12 villages/sites in Tanzania**

**Villages**	**Altitude (meters)**	**§ Catalytic model**[5]**(1)**	**§ Superinfection model (2)**	((2) – (1))*100 / (1)	**% change in Std. Err.**	**Predicted EIR**[18]
		**SCR (Std. Err.)**	**SCR (Std. Err.)**	**% change in SCR**		
Mgila	375	0.1228 (0.0139)	0.1451 (0.0116)	18	-17	39.1002
Kadando	528	0.0959 (0.0104)	0.1097 (0.0098)	14	-6	16.3467
Kambi ya Simba	746	0.0753 (0.0067)	0.0857 (0.0073)	14	6	4.7182
Ngulu	832	0.0869 (0.0077)	0.0980 (0.0080)	13	4	2.8899
Tamota	1055	0.0574 (0.0061)	0.0726 (0.0066)	26	8	0.8107
Goha	1163	0.0239 (0.0028)	0.0306 (0.0034)	28	24	0.438
Lambo	1188	0.0099 (0.0017)	0.0123 (0.0021)	25	24	0.3799
Funta	1240	0.1033 (0.0108)	0.1197 (0.0101)	16	-7	0.2824
Mpinji	1445	0.0065 (0.0012)	0.0083 (0.0015)	27	29	0.0878
Kilomeni	1556	0.0046 (0.0010)	0.0058 (0.0013)	27	27	0.0466
Kwadoe	1564	0.0071 (0.0015)	0.0092 (0.0019)	29	29	0.0445
Bwambo	1598	0.0041 (0.0009)	0.0054 (0.0012)	31	30	0.0367
Average				22		
Reversion rate		0.0139 (0.0029)	0.0426 (0.0062)			
Duration in years		72	23			

§ The models were estimated using Stata software’s **ml** command; Standard errors were approximated to 4 decimal places but actual values were used in calculating the % change.

**Table 2:** **Proportion seropositive to AMA1 and MSP1**_**19 **_**in three sites (Rabola, Bakake & Baney), Bioko, 2008**

**Age group (years)**	**Seropositive to AMA1**		**Seropositive to MSP1**_**19**_	
	**Number of participants**	**%**	**Number of participants**	**%**
<1	611	13.9	615	6.5
1 <2	292	13.4	300	8
2 <5	720	22.6	728	13.3
5 <10	938	45	934	13.9
10 <15	617	61.8	621	21.4
15 <20	275	80.7	269	33.5
20 <25	349	78.8	358	39.1
25 <35	568	76.9	566	43.6
35 <50	650	69.1	635	41.9
50+	533	65.3	528	44.9
Total	5553	50.8	5554	25.3

**Table 3:** Parameter estimates for the superinfection model, Bioko, 2008

**Parameters**	**AMA1**		**MSP1**	
	**Estimate**	**Std. error.**	**Estimate**	**Std. error.**
λ_1_	0.611	0.216	0.071	0.059
λ_2_	0.04	0.007	0.022	0.003
*r*	0.178	0.033	0.072	0.039

λ_1_ = Previous seroconversion rate; λ_2_ = Current seroconversion rate; *r* = reversion rate.

## Discussion

In this paper, a mathematical model of seropositivity to malaria antigens that allows seropositivity to be prolonged by exposure was developed. This model is important because it can produce more realistic estimates of the duration of seropositivity. This is analogous to Dietz model, which allowed for superinfection and produced more realistic estimates of the duration of infection as compared to the original Ross-MacDonald malaria model [14-17], which also ignores superinfection. However, some discrepancies may remain, considering the simplifying assumptions included in the model. In particular, the reversion rate may depend on age and the seroconversion rate may also depend on age.

The estimates of seroconversion rates at the 12 villages in Tanzania were lower than Bodker and colleague’s predicted EIR [18]. It may be that the predictive model developed by Bodker *et al.*[18] is no longer valid: the dynamics of malaria transmission might have changed and the data used for the predictive model was collected a decade (1995/96) before the current study by Drakeley *et al.,* to which the model is being applied. The estimate of the mean duration of seropositivity was lower in the superinfection model than the catalytic model but it still seems too long. One possible reason is that the model assumed a constant reversion rate but it may depend on age. For example, in a cohort study of young children, aged six years and below, conducted in The Gambia to investigate the determinants of antibody response longevity, Akpogheneta and collegues have demonstrated that antibodies decayed more slowly among children in the oldest age group and more rapidly among children in the youngest age group [19]. The superinfection model also assumes a constant seroconversion rate but it may also depend on age. For example, Nasell has shown that young children may experience superinfection up to age 5 but it is not common in adults [10]. In a multistrain model, Milligan and Downham found similar conclusions that the fraction of infection increased more rapidly in younger children than in adults as they acquire immunity to different strains of pathogens [9]. In a recent study, Portugal and colleagues held an opposite view that superinfections are uncommon in younger children [20]. A model that allowed the mean duration of seropositivity to depend on age may give a more realistic estimate of the seroconversion rate.

A better fit could probably be obtained only at the cost of complex model specification. Future work should consider extending the model to allow the reversion rate to depend on age according to an exponential function. The function can be chosen such that the duration of seropositivity is three years for adults, which is consistent with results from longitudinal studies. A model that allowed the seroconversion rate to drop gradually according to a logistic function is also an important consideration. This may be a more realistic model for how seroconversion rate changes over time than the model that assumes an abrupt change. It may not be possible to fit complex models with age-dependent duration, the parameters may not be identifiable. It may be necessary to consider the mean duration (as a function of age) to be fixed, determined from field data, and then use the superinfection model with the duration parameters fixed, to estimate the seroconversion rate, perhaps with the more flexible logistic function used to estimate trends over time. This then is a topic for further research, to find out if the mean duration can be considered constant for a particular type of antigen and it would be necessary to find reliable estimates.

## Conclusion

The superinfection model is important because it can produce more realistic estimates of the duration of seropositivity. This is analogous to Dietz model, which allowed for superinfection and produced more realistic estimates of the duration of infection as compared to the original Ross-MacDonald malaria model, which also ignores superinfection.

## Competing interests

The author declares that he has no competing interests.
